# Book Reviews

**Published:** 1819-06

**Authors:** 


### CRITICAL ANALYSIS

### OF RECENT PUBLICATIONS,

IN THE

### DIFFERENT BRANCHES OF MEDICINE, SURGERY, &c.

*Transactions of the Association of Fellows and Licentiates of the King's and Queen's College of Physicians in Ireland.* Vol. II.—8vo. pp. 602.

*(Continued from p. 424.)*

XVI.—*Observations on Varix and Venous Inflammation; with Instructions for operating, with Safety to the Femoral Vein, in Popliteal Aneurism.* By RICHARD CARMICHAEL, M.R.I.A. one of the Surgeons of the Richmond Hospital, House of Industry, &c.

THIS memoir commences with the history of a case, the subject of which was a healthy young man, where the vena saphena was tied for a varicose state of the veins of the leg, with an ulcer situated above the inner ankle, which terminated fatally: (of this, however, there was not absolute certainty; since the patient was taken away from the hospital, by his friends, when apparently dying.) In addition to the fever, and other consequences of inflammation of the vein, "four or five tumors formed on different parts of his body; on his hips, shoulders, and breast, which quickly suppurated: some of these I punctured, as soon as a fluctuation was evident, and the integuments discoloured. About an ounce, or an ounce and a half, of healthy-looking matter, was discharged from each tumor; but it was fœtid, and attended, at the same time, with a disengagement of some very offensive gas, which bubbled through the matter."

The only case on record, in which similar phenomena were observed, Mr. Carmichael remarks, is one (the 49th) related by Mr. HODGSON:

"Mr. HUNTER," says Mr. Carmichael, "was the first to call the attention of the profession to the occurrence of inflammation of the veins, which he attributes chiefly to inattention in closing the orifice of a vein after phlebotomy. Before the publication of his observations on the subject,* the untoward symptoms, which

* See *Transactions of a Society for the improvement of Medical and Surgical Knowledge*, page 18, vol. i.

sometimes occurred after venæsection, were, in general, attributed to a wound of the tendon of the biceps muscle."

Unless we go back to ARETÆUS, who treats of inflammation of the vena cava, and the symptoms that ensue from it, (*De Causis et Sign. acut. Morb.* cap. viii. l. 2,) this pathological phenomenon appears to have been overlooked, or mistaken, by the predecessors of our great physiologist; and, although observations on this subject, have been published by several eminent practitioners of surgery, as SASSE, MECKEL, OSIANDER, REIL, FRANCK, MARJOLIN, BICHAT, DELPECH, HODGSON, HOME, ABERNETHY, CHARLES BELL, BRODIE, HENNEN, TRAVERS, and BRESCHET, it is yet so imperfectly understood, that we seize with avidity any useful addition to our knowledge respecting so serious a malady.

Inflammation of the veins is believed, by Mr. Carmichael, to be of more frequent occurrence than is generally imagined; and he thinks that many lives are annually lost from this cause alone, even when its existence has not been suspected.

"Now that blood-letting is so generally practised in every description of fever," he continues to remark, "it is incumbent upon practitioners to be aware, that a train of symptoms, strongly resembling those of typhus, may arise from venous inflammation, and from which it is difficult to distinguish it; except, indeed, the inflammation of the orifice, and the pain and tenderness along the course of the vessel, may lead to a true diagnosis. In extensive wounds, or surgical operations, I believe it to be a still more common, but unheeded, cause of death; and the following case will afford a convincing elucidation of this remark."

Our limits oblige us to confine our account to an abstract of the history above alluded to.

James Boyle, æt. 40, was admitted into the Richmond Hospital, on the 20th of May, 1818, on account of a large popliteal aneurism on the right side. On the 25th, Mr. Carmichael tied the femoral artery, immediately above the part where it passes under the sartorius muscle. Some little force was required to pass the needle under the artery; and, as soon as it was accomplished, a gush of venous blood followed, which, in a second or two, spontaneously ceased. On the third day, the wound had apparently healed by the first intention. But, on the fifth day, a small quantity of pus flowed through an opening in the cicatrix. He now began to feel uneasiness: his pulse, the following day, rose to 90. His sleep was disturbed, and his countenance flushed; but he could not point out the particular cause of his uneasiness. June 7th, the thirteenth day from the operation, he had several distinct rigors, followed by increased heat; the discharge from the wound had augmented in quantity; and the lower part of the leg had become œdematous. He sighed, or rather mourned, frequently; but was still at a loss to point out any seat of pain. On the 8th and 9th, he had strong rigors, followed by profuse perspiration. His face and neck were, in general, of a deep-red colour. His manner evinced great torpor and debility; and, at times, he muttered incoherently to himself: pulse 80; tongue brown. Six ounces of blood were taken from his arm, which exhibited the buffy coat: twelve ounces more were afterwards taken on the same day. Calomel, and cathartic medicines, were given internally. The 10th, no signs of amendment: ten ounces of blood were taken from the arm, which exhibited the buffy appearance. The entire limb was now swoln and œdematous. The 11th, evidently worse: pulse, upwards of 100; tongue, brown and dry; respiration, oppressed and laborious. In the evening, he had hiccough and another rigor; after which, his face, which was hitherto of a deep-red, became pale and ghastly; and he was delirious during the night. Wine was now given to him. The 12th, the pulse 130: the integuments in the ham, covering the tumor, were livid and mortified. An opening was made in them, which set free a quantity of putrid coagulum, the contents of the sac. On the morning of the 13th, he died.

*Examination after death.*—The matter from the wound was found to have proceeded from a small abscess, behind, and on the inner side, of the artery; but, as far as this vessel was concerned, nothing was amiss. The crural vein, lying behind, and in close contact with, the artery where the ligature had been passed, had been wounded by the needle; but no portion of this vessel was included in the ligature. Its interior surface was lined with pus and organized lymph, exhibiting the appearance which membranes present in a suppurating state. This appearance extended downwards to the ham, where it suddenly ceased; but the vein was rendered impervious at this part, by a deposition of coagulated lymph. The disease also extended a considerable way down the saphena: upwards, it was traced as far as the common iliac vein. The dissection could not be carried farther, as the friends of the patient were waiting for the body; and Mr. Carmichael had been obliged to promise, that he would only examine the limb.

Mr. Carmichael, from the accident that occurred in this case, is led to make some remarks on the difficulty of passing a ligature round the femoral artery, without injuring the vein, in consequence of the close contact, and indeed adhesion, of those vessels, from the tendon of the biceps muscle almost to Poupart's ligament. The vein, he is disposed to think, is often wounded in the operation for popliteal aneurism, although inflammation may not be the result. This was found to be the case in a patient who died in consequence of secondary hæmorrhage from the artery, after that operation, which was performed by an expert and able anatomist. Such an accident has caused at least one death; and probably, others beyond number: to obviate its future occurrence is, therefore, an object of considerable importance.

After some remarks on the directions given by Mr. Hodgson and Professor Scarpa, for the mode of applying the ligature to the femoral artery in this situation, shewing their insufficiency, and want of due precision, on all occasions, Mr. Carmichael proposes that which, he believes, is not liable to similar objections.

"In a middle-sized man," he observes, "the vein begins to emerge from under the artery at five fingers'-breadth, or three inches, beneath a transverse line, ranging with the upper edge of the symphisis pubis; and is fully exposed, at four fingers'-breadth, or two inches and a half, below this line, to admit of being laid bare by dissection, so as to enable the operator to pass the needle with ease and safety between the two vessels. This spot lies considerably below the origin of the profunda and the junction of the saphena with the femoral vein: the latter, after this junction, completely emerges from under the artery; on the pubal side of which it lies in the same plane, until both vessels are concealed from our view by Poupart's ligament.

"If the pulsation of the artery is not so obvious as to direct us where to make our incision, we may err in approaching too near the pubis; and thus, independent of other consequences, embarrass the subsequent steps of the operation, by opening the saphena vein: but this may be avoided, by measuring the distance between the symphisis pubis, and the most anterior point of the spinous process of the ileum. In middle-sized male subjects, this measurement usually gives five inches and a half, and in females half an inch or an inch more: one-half of this measurement, then, brought to a transverse line from the upper edge of the symphisis pubis, will give the exact situation of the ilial side of the artery, at the place where the ligature is to be passed; which is at two inches and a half, or at the most three inches, from this point, along the groin towards the knee. In cutting down upon this spot, we come upon the pubal edge of the sartorius, where that muscle meets the artery; and here we have a strong dense fascia (the fascia lata), extending from the muscle over the vessels; but it is considerably more dense over the latter. This fascia may be divided with safety on the sartorius; and, by pursuing the dissection of it, we expose, first the artery, and then the vein; and, when the vessels are thus sufficiently exposed, we may insinuate between them, with ease, the aneurismal needle, which is to be pushed under, and close to, the artery, without disturbing it from its bed. When the point of the needle appears at the opposite (the ilial) side of the artery, we must satisfy ourselves, before we force it through, if we meet with any obstruction, that it is merely produced by cellular membrane, and is not occasioned by either the vein, or a branch of the crural nerve (saphenus), which is usually found lying in contact with the ilial side of the artery; and which is, therefore, in great danger of being injured, or included in the ligature."

Mr. Carmichael then relates a case of inflammation of the veins, after amputation of the leg above the knee, which terminated fatally. He refers to Mr. HENNEN's Work on Military Surgery, for similar cases; and is disposed to consider them as of not unfrequent occurrence. A similar remark was made by Mr. Hunter; and a case of this kind is related by Mr. Travers, in his Essay on Inflammation of the Veins. Dr. DENMAN, not long before his death, observed, in conversation, that he suspected that many cases of fever, of a typhous character, in puerperal women, arose from such an affection of the veins of the uterus. This, we consider, is an interesting subject for investigation.

The cases related by Mr. Carmichael made such an impression on his mind, that he

"Never ventured upon the general practice of tying the vena saphena, on account of a varicose state of the veins of the leg; and, until Mr. Brodie's proposition of dividing the branches instead of the trunk, was communicated to the public, I contented myself with merely recommending the use of the laced-stocking, or the application of the roller, with a view to palliate, rather than cure, the complaint."

The remainder of this very valuable memoir is occupied by the relation of the results of the experience of Mr. Carmichael in the practice of Mr. Brodie. Of this we have already given an account, in our last "Historical Sketch of the Progress of Medicine."

XVII.—*Case of incurable Disease of the Arm, arising from extraordinary Circumstances.* By the same.

A case of severe inflammation of the whole arm, arising from several needles having been wilfully driven into different parts of the limb, by the patient herself, apparently with an intention to excite commiseration in some benevolent persons, whom she attempted to deceive by her hypocrisy. The limb was amputated, when the constitution began to sink from fever and diarrhœa; and the life of the patient preserved.

The following is the only pathological observation of particular interest:

"The muscle in which the needles lay was almost changed to a firm, gelatinous, structure; and they were surrounded by firm lymph, almost of the consistence of softened cartilage; which seems to be the process employed by nature to insulate such extraneous bodies from the surrounding parts, as do not excite suppuration."

XVIII.—*On the Origin of Intestinal Worms, particularly the Ascaris Vermicularis.* By JOHN MILNER BARRY, M.D. Cork.

"The origin of intestinal worms," says Dr. Barry, "has been, for a long time, a fertile source of conjecture, and of difference of opinion, amongst medical writers. Practical writers, indeed, are too much disposed to undervalue enquiries, which are not immediately directed to the cure of the disease; alleging, in this instance, that it is the duty of the physician to expel these troublesome animals, when they are present, without being over-solicitous to ascertain their mode of production. Such reasoning is calculated to check every useful enquiry into nature, and to reduce the medical art to a state of blind empiricism."

The title of this article, and the passage we have transcribed, with which it commences, called forth our most earnest attention, and led us to expect that we should be gratified with some information of importance respecting so interesting a subject. As nothing is more easy than to pass either general praise or censure on a series of opinions, with a certain degree of security from the imputation of erroneous judgment; nor any thing less useful to the cause of science; we shall enter into the consideration of the observations and reflections adduced in this memoir, in a particular manner, before we express any opinion respecting the merits of the whole.

"It has not been satisfactorily proved," says the author, "that any of the species of intestinal worms, which chiefly demand the attention of the physician, has been derived from an external source. The doctrine of equivocal generation is now, however, altogether abandoned by philosophers."

The first of the above propositions, is the admitted truth that gives such a particular degree of interest to this subject; the second, somewhat surprised us, particularly when advanced by a physician of the learning and talents of Dr. Barry; since several naturalists and physiologists, whose opinions are considered, throughout Europe, as deserving of the highest respect on such a subject, continue to favour the doctrine of the equivocal (as it is termed) generation of some species of animals. We could adduce numerous authorities on this point; but the following will, we consider, be sufficient to shew that the question is by no means decided, as Dr. Barry has asserted.

LINK*, and REIL advocate it; so did D'OUTREPONT,† a few years since, in a work, shewing great research and accuracy of observation. This hypothesis, with respect to the origin of intestinal worms, was considered equally probable with that attributing it necessarily to ova, by one of the most learned naturalists of the latter part of the last century, RETZ. He thus expressed himself: "Ingenuè fateor unam hypothesim non minus obscuram esse quam alteram; fateor etiam me nescire, quæ vera sit harum, nec opinari me audere, ob difficultates ab utraque parte mihi impenetrabiles. Dies fortè docebit."‡ RUDOLPHI, whose opinions certainly merit respect, is still in doubt on this subject. The Theory of Nature recently published by two celebrated French philosophers, GAVOTTY and TOULOUZAN,§ favours the hypothesis of the probability of spontaneous generation, on some occasions; but, as the plan of their work did not admit of details on inferior points, their opinion on the subject immediately under consideration is not advanced.

As Dr. Barry's observation applies only to living philosophers, we cannot adduce the opinions of those who do not exist; and, therefore, authorities of the first importance respecting this question must be passed over unnoticed.

It should not, from those citations, be imagined, that we express any particular opinion in favour of casual generation: they are merely adduced to shew, that the assertion of Dr. Barry is rather dogmatical, than deduced from an unbiassed view of the subject. No person, in his senses, expects to see

> "—— ubi desuerit madidos septemfluus agros

> Nilus, et antiquo sua flumina reddit alveo,

> Aëthereoque recens exarsit sidere limus;

> Plurima cultores versis animalia glebis

> Inveniunt; et his quædam modo cœpta, per ipsu

> Nascendi spatium: quædam imperfecta, suisque

> Trunca vident numeris; et eodem corpore sæpe

> Altera pars vivit; rudis est pars altera tellus.

But the arguments in favour of the probability of the formation of hydatids, and other vermicular animals, from

---

* *Versuch einer Geschichte und Physiologie der Thiere.* Chemnitz, 1805.

† *Perpetua Materiei Organico-Animalis Vicissitudo.* Halæ, 1798.

‡ *Lectiones Publicæ de Vermibus Intestinalibus, imprimis Humanis*, p. 55. Stokolmiæ, 1788.

§ *Essai sur l'Histoire de la Nature.* Paris, 1815.

the coagulable lymph of the blood, have not been satisfactorily refuted.

Montesquieu* remarked, respecting an analogous phenomenon, the growth of *fungi*, and some other species of cryptogamous vegetation:—"Cette opinion (l'origin des mousses des graines) est fondée sur une raison de commodité; et chez bien des gens, cette raison supplée à toutes les autres." If Dr. Barry had seen a little of military hospitals during an active campaign, and witnessed the excrescences of the fungous species, (somewhat like champignons,) growing from the external surfaces of bandages of wounds long neglected,—a phenomenon, we believe, first observed at the Hôtel-Dieu, by Mery, and then attributed to the vinegar used diluted as lotions; but since determined to arise without the presence of any vegetable substance, except the linen bandages, &c.; he would, perhaps, have taken a different view of the nature of organization.

There are many observed phenomena, which favour the hypothesis that there is sometimes originally produced, from the matter of living vegetable and animal bodies, (by a deviation from the ordinary influence of the organizing principle,) vegetables and animals of a different species from those whence they derive their origin; and which will continue to propagate themselves in the manner observed in polypi, and several cryptogamous vegetables. These, it must be evident, on this hypothesis, must be very simple in their organization; and, therefore, the objection that has been made to some other views of this subject,—that we do not gather grapes from thistles, is not really applicable to it. The nature of a review will not permit us to enter into details; but such of our readers as are naturalists will readily recognize the phenomena to which we allude.

Returning to Dr. Barry, we find he observes, that

"The lumbricus ascaris, and the lumbricus terrestris, were for a long time, and are still, properly referred to the same species; and, though a few obvious distinctive marks had been discovered by some of the earlier physicians and naturalists, particularly Willis and Ray, it was reserved for modern times to remove all doubts on the subject. That accurate anatomist and eminent physician, Dr. Baillie, having compared the internal and external structure of these animals, has pointed out many circumstances,

---

* *Observations sur l'Histoire Naturelle*, obs. v. There are some curious and interesting observations on this subject in Bacon's *Sylva Sylvarum*, cent. vi.; which may be consulted with profit as well as pleasure.

which prove that they are not of the same species;* and, since the publication of the Morbid Anatomy, Dr. Hooper has, by a most minute investigation, established the distinction between these worms beyond dispute."

We have taken some pains to discover the author's meaning, in the above paragraph, but without success: "*Davus sum, non Œdipus.*" We shall therefore pass on to what is at least intelligible.

"The *ascaris vermicularis* is also still generally considered peculiar to the human body; as no well-authenticated facts have yet been published, which prove that this species has been found to live and grow in any other medium. In the sequel of this essay, I shall prove the fallacy of the opinions hitherto entertained respecting this worm; and shew, by undeniable facts, that it is derived from without."

The following is an abstract, omitting nothing but adventitious and unimportant circumstances, of the account given of those facts by Dr. Barry:

Mr. H— and his family became affected with *ascarides*, soon after they went to reside near Macroom, in the county of Cork, where Mr. H— had built a house; near which was a spring of water, that supplied the family for drink, &c. Strangers, visiting Mr. H—, were sure to become affected with ascarides. After leaving this place for a short time, Mr. H— and his family were greatly relieved from these worms; but they re-appeared, on their returning to their usual place of residence. Mr. H— was then induced to quit it; and his flight was accelerated by a discovery, made by Mrs. H—, of myriads of dark-coloured worms, in the water of the spring on which their suspicions had been fixed, which resembled in every respect, except in colour, the ascarides they were accustomed to pass from their bowels. Dr. Barry examined some of those animalcules.

"They varied in length, from half an inch, and under, to nearly three-quarters of an inch, tapering gradually from the head to the tail, which ended in a point. They were proportionably different in bulk; the largest being as thick as a stout pack-thread, and the smallest so minute as to be scarcely visible without the help of a magnifier: with others, of all the intermediate degrees of size. The colour of the largest worms, and those of a middle size, was dark-brown, when taken from the well; but, upon wiping them gently with a napkin, the colour changed to a very pale yellow; of which colour were numerous small worms, some of which, as I have stated, were visible only by the help of a magnifier.

"Upon comparing the worms of the well with those discharged from the bowels, the resemblance was exact, in shape and external

---

* Vide Baillie's *Morbid Anatomy*, and Hooper on *Intestinal Worms*. Mem. Lond. Med. Soc. vol. v.

appearance. The largest worms from the well exceeded in size those passed from the body, but not remarkably; and they differed also, in being dark-coloured. This difference of colour may be urged as an objection to the common origin of the ascarides from the well, and those of the intestines; but we have the authority of Hooper for the fact, that ascarides, of a brown colour, are sometimes discharged from the body; and there are numerous instances to shew, that animals, as well as vegetables, become light-coloured by immersion in darkness."

The identity of the worms of the spring with those passed from the bowels of Mr. H— and his family, is very probable; but we concur with the "learned naturalist," to whose opinion Dr. Barry alludes in the following passage: —"A learned naturalist, who has done me the kindness to peruse these papers, objects, that the ascarides of the well may have been of a different species from those which occur in ordinary cases; and alleges, that, to render the proofs perfect respecting the identity as to species, of the ascarides of the well, with those discharged by Mr. H—, (which he allows to be the same,) and the ordinary intestinal ascarides, they should be minutely compared in their anatomical structure:"—and we are surprised that Dr. Barry did not follow the judicious suggestions of his friend. He might, then, have avoided the unfortunate dilemma into which he has fallen, of advancing mere notions as positive truths; and then adducing, with the view to confirm them, observations which appear utterly inadequate to afford them the smallest support.

That we may pass over nothing which Dr. Barry considers tends to support his opinion, we shall transcribe his concluding observations:

"Mr. H—, and his family, have never been wholly free from these worms, during the years which have elapsed since he was driven from his former residence; though their numbers have never been so great, nor the sufferings of him and his family so intolerable, as when they resided near the prime source of the mischief."

Before we dismiss this subject, it may be proper to remark, (since Dr. Barry seems disposed to apply his observations in a general manner,) that, even were the identity of the common *ascaris vermicularis* with those of the spring near Macroom determined, it would afford but little or no basis for positive opinions respecting the origin of the *tænia*, and *vesicular worm* or hydatid, of the human body; which will not admit of a comparison with any other organized being, existing without other animal bodies. There is, in the cabinet at the university of Pavia, a tape-worm above 230 feet in length. Now, admitting that the abundance of nutriment furnished in the intestines of other animals may occasion an extraordinary growth of the worm, still we should expect to find it, elsewhere, so large as to be at least visible; and that it would not have escaped the researches of naturalists, were its formation not essentially dependant on the secretions from the intestines. It may, perhaps, be said, that these secretions are merely a necessary and appropriate nidus for the development of the ova: were it so, we should expect to find the worms similar in structure in all the animals in which they exist; which is not the case.

Some naturalists have thought, that they had effected a great deal in favour of generation from ova, by discovering worms, somewhat similar to those most common in the human body, in other animals; and thus making it appear, that we thence received them with our food. How did they originate in those animals? All the difficulties attendant on this subject are, however, got rid of, by the hypothesis of Vallisneri:* he says, they existed in the first-created human beings; and have been, successively and uninterruptedly, transmitted from mother to child, through the medium of the blood of the placenta! The hypothesis of Bonnet, respecting the generation of human beings, is now generally judged to be irrational: but, if this be considered as an aberration of the imagination from the bounds of truth, what is that of Vallisneri?

Dr. Barry says, "I think it unphilosophical to deny the extrinsic origin of worms; because there is, as yet, no actual evidence of their existing, and attaining their usual size, except in animal bodies." This sentiment is very just: but it is still more unphilosophical, positively to assert their *extrinsic origin,* without the knowledge of any facts which precisely support such an opinion. Bacon would teach us to hold our judgment in suspense, in the present state of our knowledge of this subject: he would say, Man "tantum facit et intelligit, quantum de naturæ ordine observaverit, nec amplius scit aut observare potest."

The rest of this volume is occupied by Reports of the *Fever Hospitals* in Dublin. Of these we shall communicate to our readers only a partial account, as we have already, in different parts of our Journal, treated somewhat in detail of the nature and character of the epidemic fever of that city. A great proportion of these Reports is taken up with

---

* *Opere Fisico-Mediche.* Venezia, 1733.

observations respecting the origin of the disease; remarks on prophylactic measures; and the appropriate arrangement of fever-hospitals, &c.; with much matter of mere local interest: of which, it is obvious, a general view would be of but little or no utility: and our limits will not permit us to enter into further details on the present occasion. We shall, therefore, confine our observations to what relates to the pathology of this epidemic fever. It is this which will be the most interesting, as well as useful, to the greater proportion of our readers.

XIX.—*Medical Report of the Fever-Hospital, and House of Recovery, Cork-street, for the Year* 1816*: to which is added, some Account of the succeeding Epidemic, exemplified by Cases lately admitted into that Hospital.* By WM. STOKER, M.D. Physician to that Hospital, and Licentiate of the King's and Queen's College of Physicians, &c. &c.

Dr. Stoker is disposed to adopt the following division of inflammatory fevers, considering it as simple, and applicable to practical utility:—"1st, Idiopathic fevers, comprehending those of spontaneous and contagious origin; 2d, Inflammatory fevers; and, lastly, those in which these two kinds co-exist, or mingle together." We suppose, Dr. Stoker means to signify, when a febrile state exists alternately with and without local inflammation; not where idiopathic and inflammatory fever *co-exist.* By inflammatory fever he signifies that which, he considers, is really dependant on local inflammation. He continues to remark:

"The opinions on which this division rests, that typhus and synochus are not essentially inflammatory, but that, in their simple forms, are diseases of debility through their whole course; and that the excitement, so observable in their early stages, is constitutional re-action; accord with my experience."

As this question respecting the nature of Fever has already occupied much of the space of our Journal in the late numbers, we shall not, on the present occasion, enter into particular arguments on the subject; yet we must observe, that it is with extreme regret we witness such opinions still advanced by physicians of eminent talents; for, we not only believe the doctrine, that febrile phenomena are merely consequences of local disease, and that of an inflammatory nature, to be one of the best substantiated points in physiology; but we also think, that the admission of that doctrine, is a principle of the first importance in the practice of medicine, as well as in the theory of diseases in general. From the time this doctrine was first advanced in England, by Dr. PEW,* it has, however, been gradually gaining ground; and is now advancing with rapidity and firmness, acquiring additional vigour as it proceeds in its course.

This is not one of those hypotheses which has been produced in a day, to hold a short dominion over minds solely influenced by enthusiasm, and then to sink into oblivion; of which we yearly witness the rise and fall of at least one, in a certain nation in Europe, since the doctrines of BROWN gave place to chemical materialism, philosophical idealism, and mystic fanaticism: it is the result of meditation on facts illustrated by the brightest truths in physiology; and its progress has been, like that of all great and important truths, slow and gradual in the commencement of its career, and cautious and reserved in its efforts, until it had encountered and withstood the opposition of conflicting opinions. But, as it is our desire to elicit truth, not to gloss over particular opinions, we shall adduce the most important observations of Dr. Stoker in favour of his own views:

"Morbid anatomy," he observes, "therefore, does not appear to me to warrant the conclusions, which those who hold the opinion of typhus fever being an essentially-inflammatory disease, have deduced from it. I have, in some instances, observed the same partial turgescence of vessels which they report, and likewise signs of inflammatory action, in various parts of the bodies of those who died of fever. The former, however, I believe, is by no means a mark of previous inflammation; and I could generally trace the commencement of the inflammatory action, which produced the latter appearance, to local disease, which preceded or supervened on fever, sometimes at late stages. But, in several cases, where I had witnessed the highest degrees of febrile excitement before death, no such signs of turgescence, or of inflammation, were observable on dissection."

In order to give any importance to the remarks contained in the above paragraph, Dr. Stoker should have explained what are his ideas respecting the "turgescence of vessels;" and what is the nature of the "local disease" which precede fever, and produce inflammation. Arguments might then, perhaps, have been adduced in opposition to his opinions, and truth thus elicited; but, in the manner in which they are here expressed, they are secure from any direct attack. With respect to the statement, that the local inflammation supervened on fever, sometimes at late stages; that is a gratuitous assumption. We, however, do not deny that secondary local inflammation does occur during

---

* *Medical Sketches*. 8vo. London, 1785.

the paroxysms of excitement in fever. The brain appears to be frequently affected in this manner; but this avails nothing with respect to the subject of dispute. We have had frequent occasion to witness the existence of inflammation of the mucous membrane of the stomach and small intestines, to such an extent as to terminate in ulceration and gangrene, without having been attended with pain; indeed, it requires the presence of very acute inflammation of that tissue, as well as of the heart and other parts chiefly supplied with nerves from the ganglionic system, to produce pain: and it is in consequence of this, that disease of those organs is so frequently undiscovered until after death.

The case of inflammation of the femoral vein, which we have noticed in Mr. Carmichael's paper, in the present volume, will illustrate this point. Here was inflammation existing to such an extent, as finally to cause death, without producing pain; and which was also accompanied, almost in the commencement, with all the ordinary symptoms of "*typhus gravior*." Had such a case occurred to the observation of those who have adopted the opinions we oppose, what idea would they have entertained respecting its nature, and how would it have been treated? It would, doubtless, have been termed *idiopathic fever*, arising from debility; treated, probably, by stimulants; and then, when death had shewn the existence of inflammation, this would have been called *consecutive*. This is not a solitary case with respect to its character; it is the common result of inflammation of the veins: it is similar to what occurs in almost all instances of inflammation confined to parts subservient to *organic life*, which are not, for especial purposes, supplied with nerves from the spinal marrow or brain, as well as from the ganglionic system. When inflammation of these parts is, however, very acute, the whole system of the same nature becomes secondarily affected; and, as some of its organs (the stomach, for instance,) are well supplied with cerebral nerves, the brain is consequently excited, and a new character is thus given to the disease. We may be taxed with running into mere hypothesis on this subject, and thus falling into the error we have just censured; but, what has here been advanced, naturally flows from reflection on the best-established points in physiology, and from observation of what actually occurs in disease. Although we point out the *Histoire des Phlegmasies Chroniques* of M. Broussais as an excellent medium in which these facts may be contemplated in default of sufficient sources for original observation, and for the most rational notions that may thence be deduced, we know that the mode in which that physician has seen facts, and the inductions he has thence formed, are liable to the very objections we are ourselves about to advance respecting the conduct of others. But, amidst the fallacy of the testimony of the senses and the decisions of the human understanding, we must admit those opinions that appear to be best supported.

With respect to the proposition, with which the paragraph last cited from Dr. Stoker concludes, we venture to remark, (but assuredly without intending any personal allusion to Dr. Stoker, whose talents we respect,) that the most decided appearances of inflammation have been detected in bodies, by those whose views pointed out where it should be sought for, when it had been passed over undiscovered by other investigators of perhaps equal general talents. This is very common in researches in science: men only see those objects which are situated within the compass embraced by their particular views. How much did MORGAGNI perceive, that would probably have been as though it did not exist, to any other observer?

Dr. Stoker inserts a letter from Mr. KIRBY, which, in some degree, illustrates what we have stated. "The brain," says this distinguished anatomist, "*so constantly supposed to be the seat of inflammation* (in fever), rarely exhibited the characters indicative of such a state. There was seldom any evidence that the peritoneum, or abdominal viscera, had been the seat of local inflammatory action." We imagine, that Mr. Kirby's researches were too much influenced by an idea, that the hypothesis of inflammation of the brain being the sole cause of fever was generally admitted; and that, having shewn the want of foundation for that hypothesis, he had effected what would be considered a circumstance of the highest importance, relative to the disputed points on the nature of fever. Not a word is said expressly of the *mucous membranes*. How frequently have anatomists, eminent for the possession of talents, been known to look at the outside of the abdominal viscera on these occasions, and seeing there no marks of disease, say, None exist in these parts?

It should be borne in mind, that Mr. Kirby's observations were made on bodies which had been interred, and afterwards taken up and brought to his anatomical theatre for dissection; they must, consequently, have been made some days after the extinction of life: at this period, what are considered as the decisive signs of inflammation will frequently have ceased to be evident, when it is the superficial membranes, especially the mucous tissue, that have been the seat of the disease. If the fever have been of long duration, these signs will, indeed, not be very strongly marked, in some instances, immediately after death: the inflammation of those membranes may have subsided, after having produced effects on the brain, or other organs, that will be fatal; and such effects will ensue in many states of the animal economy, without any considerable degree of lesion of the parts primarily affected. The work of M. Broussais, to which we have referred, and which he terms a monument of his ignorance, furnishes numerous incontestible evidences of the truth of these statements.

The functions of the mucous tissue, the changes which take place in it from disease, and the influence of this on the rest of the animal economy, have not been sufficiently studied by the generality of physicians. Many seem to expect that inflammation of that membrane should be attended with pain, and that when it has been so intense as to produce fatal effects, that it should have given rise to the formation of purulent matter, or ulceration; neither of which are necessary, nor indeed very frequent, results.

There is another circumstance that must not pass unnoticed: it does not appear that the spinal marrow was examined by Mr. Kirby. Dr. Sanders, of Edinburgh, has for many years taught and demonstrated the importance of such an investigation, in researches respecting the nature of fever, as well as in a variety of other forms of disease.

False conclusions are frequently deduced from the notion, that debility of the capillary vessels of a part will give rise, primarily, to an accumulation of blood in it; that these vessels will become passively dilated from the impulse given to the blood by the heart. Thus, we frequently hear of congestion of blood in the capillaries, from debility. This is one of the remaining errors deduced from the mechanic and hydraulic hypotheses; in which the heart was considered as the sole agent of the circulation of the blood. More accurate attention to physiological phenomena, whether of health or of disease, would shew, that, when the capillary vessels are in a state of debility, they are always collapsed; and the parts supplied by vessels in such a state, are universally shrivelled, cold, and exsanguinous. In order to preserve the transmission of blood to them, the capillaries must be in a state of active dilatation, in a sort of erethism, they must call it, in a manner, to use a metaphorical expression of Bichat: without this, the blood will not be sent to them; they will collapse, not suffer distension.

Let us express this in another way:—The influence of the nerves on the capillary arteries causes an erection and a dilatation of them; they thus form open capillary tubes, into which the blood rushes: without nervous influence, they lie flattened, and such a degree of *vis à tergo* as can be exerted by the heart would increase or promote this obliteration of their cavity, by pressing the sides of the vessels together, after the larger ramifications had become distended.

As the chief observations and reflections of Dr. Stoker, respecting the pathology of fever, and on the mode of treatment, are consonant with, and deduced from, those we have already noticed, we shall pass over the remaining part of this paper; since, our remarks on it would occupy space we are disposed to devote in a manner more useful to our readers; and our duty, as reviewers, would not permit us to insert in this part of our Journal, without remarks, such opinions as are not consonant with those we have been led to adopt from our own observations, and from reflection on the doctrines of the majority of the most eminent of existing physiologists. Such an abstract as our limits would permit us to adduce, would convey but little idea of the more valuable part of this memoir; and that it contains much valuable matter, our readers will readily be induced to believe, notwithstanding what we have said respecting it on some few particular points. The productions of all men of genius must receive such a character, even from those who do not agree with them respecting some ideas which may have influenced their observations.

XX.—*Medical Report of the Sick-Poor Institution, for the Year* 1817. By JOHN O'BRIEN, M D. Fellow of the King's and Queen's College of Physicians in Ireland.

We have already had occasion to speak of the talents of Dr. O'Brien; and, although we then, as we must now, noticed his opinions in a brief manner, we hope it was done in such a way as clearly to express our sentiments respecting them.

This Report, although especially devoted to observations and remarks on Fever, embraces a general view of the principal diseases affecting a district of the city of Dublin, comprising a population of upwards of fifty thousand persons. The observations are, necessarily, so numerous and various, as not to admit of an abstract. We shall, however, transcribe the following very interesting remarks: they indicate truths, until lately too generally overlooked, but which are of the highest importance in the practice of medicine. The first relate to dysentery, which was at one period epidemic in Dublin:

"The disease, which was evidently produced by the bad quality of the bread and other food of the poor, was attended with strong inflammatory fever, and severe tormina of the bowels, which very nearly approximated to enteritis. In all cases, therefore, of unusual severity, I had recourse to venæsection at the commencement, which was repeated until the tormina became mitigated, and the pulse indicated a remission of inflammatory fever. After blood-letting, the remedy which produced the most remarkable benefit, was a powder, composed of ten grains of Dover's powders and one grain of calomel, taken twice, or sometimes thrice a-day, according to the exigency. During the use of these powders, a purging-draught, consisting of infusion of senna and sulphat of magnesia, was given every second day, until the scybalæ disappeared. In addition to the above, fomentations with diluted spirit of turpentine, semicupium, and flannel bandages around the belly, were sometimes directed; but the powders above mentioned were thought to give the most substantial relief."

"Although dyspepsia frequently exists as a primary disease, arising from slight inflammation and thickening of the coats of the stomach, in which ulceration, and even erosion, have been detected by dissection; yet I believe, in general, it is symptomatic of diseases of the surrounding viscera, and chiefly of the liver. To the unfortunate habit of drinking ardent spirits, the frequent occurrence of this disorder is chiefly to be attributed; although, I am convinced, the quality of the food has also some effect in producing the disease. The dyspepsia of the poor, and indeed of the rich, arises generally from induration of the liver; and from the same cause arise the numerous instances of anasarca and ascites, to be met with among the poor."

That we may not appear eager to adduce such opinions as are favourable to the doctrine respecting fever to which we are converts, and disposed to shun the disclosure of those of men, whose talents command respect, which are in opposition to it; we shall observe, that Dr. O'Brien expresses his "dissent from the opinion that this disease (the epidemic fever of Dublin) is to be considered as one of the phlegmasiæ, and our treatment guided by this hypothesis."

XXI.—*Medical Report of the Fever-Hospital, Cork-street, Dublin; containing an Account of the Progress of the present Epidemic.* By F. Barker, M.D. Honorary Fellow of the King's and Queen's College of Physicians; Professor of Chemistry in Trinity College, Dublin; and Senior Physician to the Hospital.

Of this Report an abstract could not possibly convey an adequate idea. It is itself a condensed account of very extensive series of observations, especially on what may be considered as the more remote or predisponent causes of fever. Dr. Barker considers, in succession, the circumstances which appeared to be connected with the origin of the epidemic ; the development and progress of the malady in different parts of the country ; its contagious origin ; the influence of season on its character ; the age and condition of its principal subjects ; its immediate and concurrent causes ; the symptoms of the disease in general, and in individual cases ; the evidence in favour of critical days ; its mortality at different periods ; its essential character ; the mode of treatment ; a general view of the progress of epidemic fever in Europe in modern times, and the probability that, "thus excited on the continent, (by the influence of a state of war,) it has been introduced into these countries, (Great Britain,) where, from the operation of various exciting causes, it has become extensively epidemic ;" and the appropriate prophylactic measures. This account will be perused with much pleasure and advantage in the detail. The numerous observations are arranged with that order and precision, which shew them to be deduced from extensive views, directed by a mind well qualified to distinguish the essential from the accidental circumstances of things.

Medical logic does not, in reality, extend beyond the history of facts, and therefore to contribute to our knowledge in this respect, is to develop the principles for the practice of the healing art ; yet, we cannot help wishing that Dr. Barker had indulged more in reflections illustrative of these facts, particularly on those relative to the nature of the fever under consideration : they would, it cannot be doubted, have been of considerable utility to the medical practitioner ; and they would also have given much additional interest to the observations he has here adduced.

Here we close this volume, as far as regards the consideration of it in our official capacity ; but it will often be referred to in our private meditations. On turning to the observations respecting it which have occupied our pen, we perceive that, in extent, they really bear a due relation to the time they have engaged ; but, on revising them in detail, we discover how much important information has been left unnoticed, and how inadequately we have expressed, in direct terms, the sense we have of the value of the source whence they were derived. To do this would, however, be superfluous ; for the extracts which have here been given, are themselves the best, as well as the most appropriate, heralds of the work.

*A Practical Treatise on the Morbid Sensibility of the Eye, commonly called Weakness of Sight.* By JOHN STEVENSON, Surgeon-Oculist and Aurist to H. R. H. the Duke of York, their R. H. the late Princess Charlotte, and the Prince Leopold of Saxe-Cobourg; Member of the Royal College of Surgeons, &c.; Lecturer on the Anatomy, Diseases, and Operations, of the Eye and Ear; and Author of a "Practical Treatise on Cataract." Third Edition. 1819.

THE length of time during which this valuable tract has been before the public, and the extent of its circulation, preclude the necessity of any present comment on its general character. The views developed in it stand now confirmed by the concurrence of lengthened observation; and the author, in his more extended experience, finds no ground to depart from the opinions which he at first promulgated. No change has been introduced into the body of the work, in this third edition; but, in a preface, Mr. Stevenson has introduced some remarks, deduced from a wider range of practice in the disease, of great practical utility.

He finds that there are some unfrequent instances, where the morbid sensibility of the eye will not give way to the moderate depletion which he considered at first to be quite efficient in the removal of the disease. Some few cases have occurred to this gentleman, wherein he has been obliged to pursue the system of topical detraction of blood to an extent apparently greatly disproportioned to the indications of the disease, and yet followed by eventual success. Such cases the author looks upon as connected with a morbid state of parts more deeply seated than the immediate organ of vision, which state, perhaps of long standing, and interesting probably a considerable extent of structure, is less susceptible of influence from superficial remedies.

Some points in practice, valuable, as founded on enlarged experience, are noted, in touching on the respective influence upon diseased states of the eye, of the several modes of abstracting blood. Mr. Stevenson gives it as his opinion, that, in all the forms of acute ophthalmia affecting the external parts of the eye, the most prompt and decided relief is obtained by drawing blood from the external jugular vein. This seems, in many instances, to be alone sufficient for the removal of disease. In iritis, and acute inflammation of the deeper seated parts, the author recommends to open the trunk of the temporal artery.

We think the practical matter subjoined in this preface to the present edition, adds considerable value to a work of previously admitted merit.

*An Analysis of the Subject of Extra-Uterine Fœtation, and of the Retroversion of the Gravid Uterus.* By JOHN KING, Esq. 8vo. pp. 176. Burks and Co. Norwich; and Callow, London. 1818.

THE most interesting part of the work which we now introduce to our readers, is an account of a new operation for the delivery of an extra-uterine fœtus: to this we shall, therefore, devote our chief attention, passing over some preliminary reflections respecting the act of conception, with which it commences, (which we rather wish the author had not indulged in,) to what will render his memory identical with the history of the medical art.

The case which we are about to relate occurred in Edisto Island, in South Carolina, where Mr. King formerly resided. Nothing is said respecting the state of the patient during the time of gestation, previous to the commencement of the pains of labour, which the woman had experienced for four days, when Mr. King was requested to visit her. These pains occurred in the manner of those of natural labour, but were not attended with any other of the ordinary signs of that process. On examination *per vaginam*, the os uteri could not be discovered. The nature of the case was readily discerned by Mr. King; and, the patient being exhausted by the pains she had suffered, he, after a little reflection, determined to perform the operation, of which we shall give the history in his own words:

"It consisted in laying the vagina open to a great extent. The head of the fœtus floated and vacillated on the right side of the uterus, and pushed the uterus from its situation. I introduced a small bistoury, guarded by the end of my finger, as far as I possibly could, so as completely to embrace the circumference of the head, and thereby prevent any laceration of the parts in the progress of delivery. I then pierced the vagina through, and carried the knife five or six inches downwards and backwards, so as to insure the easy extraction of the child's head. The instant the vagina was laid open, the waters flowed abundantly; the membranes being laid open with the same incision. I then introduced my hand through the wound in the vagina, and found the infant very high up, and firmly fixed, without any prospect of its descending into the pelvis.

"As we could derive no help from the contraction of the uterus in this case, and all the efforts of the mother depending on the contraction of the recti, transverse, and oblique, abdominal muscles; I therefore desired the assistants to press gently and constantly upon the abdomen, and to imitate a circular descending motion with their hands. The mother, animated thereby with the prospect of delivery, redoubled her efforts; and, with the help of the vectis, I perceived the head to advance by slow degrees into the pelvis, and I afterwards, with the forceps, completed the extraction, after a long and uninterrupted exertion. The child appeared to be still-born; but, not being certain of that, I inflated the lungs, and was pleased to find it had borne the brunt of the day without a fatal event. The hæmorrhage from this large incision was inconsiderable. The infant was of the common size, and well-conditioned. The placenta was uncommonly small, and the funis umbilicalis remarkably thin, so that it ruptured on the evolution of the infant, though without any hæmorrhage.

"The morning after delivery, I extracted a full bleeding from the arm, and repeated an anodyne. I left the patient without complaining. I had caused her to lie on an inclined plane, upon her back, with the head very low.

"I was not able to see her again until the third day. I then found her state uncomfortable, with pain over the pubes; and, on examination *per vaginam*, discovered the intestine pushing at the wound, the wound itself being much contracted. I ordered her to lie on the left side, with the hips more elevated, to favour the retraction and gravitation of the intestines from the wound. This uneasy position favoured our views and answered our expectations. I caused a blister to be applied over the pubes, and prescribed a saline anodyne mixture, to be taken three or four times a-day. I made it an object to constipate the bowels for ten days, until the danger of any hernial protrusion was over.

"In two weeks this woman, without my consent, walked about. I then found the intestine could no longer protrude through the wound, under any circumstances of posture. In two weeks more, I could not discover that there had been any incision made in the vagina. The uterus resumed its natural site."

A similar operation has since been performed by Dr. Delisle, of Valogne,* but in this case neither the mother nor fœtus survived. This event may, however, be probably attributed to the great sufferings experienced by the mother during the whole period of gestation, which had rendered her state of body extremely feeble. She also experienced a considerable degree of hæmorrhage before the placenta could be extracted; and she was almost exhausted by labour-pains before Dr. Delisle was called to visit her. The infant was born alive, but died in about half an hour: it was not apparently of above six months and a half, or seven months.

Many circumstances render this operation preferable to an incision in the abdominal integuments, in cases of extra-uterine gestation; particularly, the less probable danger

---

* *Bulletin de la Societe Medicale d'Emulation.* Mai 1818. *Journal Universel des Sciences Med.* t. x.

from a wound in the vagina than from one in the abdomen, and the free exit that in the former situation would afford to any hæmorrhage that might ensue on the separation of the placenta. But, in opposition to this may be advanced, the greater difficulty in the extraction of the fœtus through the pelvis, when nearly full-grown, than through an opening in the abdomen. Some degree of prejudice seems to exist against the latter method, apparently in consequence of its forming part of an operation of a very different character, that of hysterotomy. It does not seem to have been sufficiently considered, that the principal danger in this case depends on the incision of the uterus, not on that of the external integuments.

The Signori Rossi, of Parma, a short time since, performed the incision of the abdomen in a case of rupture of the uterus occurring during labour, with a favourable result to the mother. The infant was, however, born dead, apparently in consequence of the placenta having been long separated from the uterus ; as the aid of those surgeons was not required until after the lapse of six hours from the time of the accident.

Extra-uterine pregnancy is, apparently, no uncommon occurrence, as a great number of cases have been recorded within these few years past ; and the propriety of performing one of the above operations without delay at the proper epoch, cannot possibly be doubted. Experience alone, we think, can determine which is the preferable method in the generality of cases. The period at which it should be executed is indicated by a circumstance, observed in nearly all the cases on record, which must confound the reasonings of the mechanists on the physiology of the human body : we mean the occurrence of uterine contraction and pains, similar to those of ordinary labour, about the period of the full growth of the fœtus. Of the laws on which this depends, we shall probably for ever remain ignorant. The causes of the enlargement of the uterus, and the formation of the floculent membrane in it, similar to the epichorion, which has also been witnessed in the cases where opportunities for examination have occurred, are more easily intelligible.

We shall not enter into a consideration of the arguments of Mr. King to prove the occurrence of extra-uterine fœtation, as we do not believe it possible that any doubts can be entertained respecting it at the present period. Those arguments would have possessed more importance a few years since, than they do now after the discoveries that have recently been made, and generally promulgated, respecting the subject to which they relate.

