# Errata

**Published:** 1819-07

**Authors:** 


In the review of a *Memoir on Intestinal Worms*, by Dr. Barry, in the last volume of this Journal, a passage was transcribed, (page 499,) in which the sense was observed to be obscure: this, we have been informed by Dr. Barry, depends on a typographical error. By reading, in the second line of that passage, *popularly* instead of *properly*, the sense of the whole paragraph is clearly developed.

